# Development of VEGF-loaded PLGA matrices in association with mesenchymal stem cells for tissue engineering

**DOI:** 10.1590/1414-431X20175648

**Published:** 2017-08-07

**Authors:** A.R. Rosa, D. Steffens, B. Santi, K. Quintiliano, N. Steffen, D.A. Pilger, P. Pranke

**Affiliations:** 1 Laboratório de Hematologia e Células Tronco, Faculdade de Farmácia, Universidade Federal do Rio Grande do Sul, Porto Alegre, RS, Brasil; 2 Programa de Pós-graduação em Ciência dos Materiais, Universidade Federal do Rio Grande do Sul, Porto Alegre, RS, Brasil; 3 Programa de Pós-graduação em Ciências Biológicas: Fisiologia, Universidade Federal do Rio Grande do Sul, Porto Alegre, RS, Brasil; 4 Programa de Pós-graduação em Ciências Biológicas: Neurociências, Universidade Federal do Rio Grande do Sul, Porto Alegre, RS, Brasil; 5 Irmandade Santa Casa de Misericórdia de Porto Alegre, Porto Alegre, RS, Brasil; 6 Instituto de Pesquisa com Células-Tronco, Porto Alegre, RS, Brasil

**Keywords:** Emulsion electrospinning, VEGF, Stem cells, PLGA, Biomaterials

## Abstract

The association of bioactive molecules, such as vascular endothelial growth factor (VEGF), with nanofibers facilitates their controlled release, which could contribute to cellular migration and differentiation in tissue regeneration. In this research, the influence of their incorporation on a polylactic-co-glycolic acid (PLGA) scaffold produced by electrospinning on cell adhesion and viability and cytotoxicity was carried out in three groups: 1) PLGA/BSA/VEGF; 2) PLGA/BSA, and 3) PLGA. Morphology, fiber diameter, contact angle, loading efficiency and controlled release of VEGF of the biomaterials, among others, were measured. The nanofibers showed smooth surfaces without beads and with interconnected pores. PLGA/BSA/VEGF showed the smallest water contact angle and VEGF released for up to 160 h. An improvement in cell adhesion was observed for the PLGA/BSA/VEGF scaffolds compared to the other groups and the scaffolds were non-toxic for the cells. Therefore, the scaffolds were shown to be a good strategy for sustained delivery of VEGF and may be a useful tool for tissue engineering.

## Introduction

The fundamental concept of tissue engineering (TE) involves the association of three basic requisites: 1) cellular therapy, in which cells are planted in a damaged tissue or in the circulatory system; 2) bioactive agents, including signaling molecules and growth factors; 3) use of scaffolds for cell support. These scaffolds allow the permeation of nutrients and the elimination of residues, as well as the localization of the cells on the lesion site (1).

In general, the substitution of damaged or non-functioning tissue through the use of tissue developed *in vitro* requires the concomitant formation of new blood vessels that are able to provide oxygen and nutrients for the neoformed tissue, a phenomenon called angiogenesis (2). Various strategies are currently in use with the aim of increasing the vascularization of the implants used in TE (3-6). Notable among these is the addition of growth factors (GFs), such as vascular endothelial growth factor (VEGF) in polymeric biocompatible and biodegradable matrices (4,7). The sequence of events during angiogenesis includes the dilation of blood vessels through the movement of the endothelial cells and the action of the proteolytic enzymes together with cellular migration and proliferation. This process is mainly controlled by the different VEGF isoforms (8). Therefore, because of the great importance of angiogenesis in TE, the present study aimed to produce polymeric scaffolds with incorporated VEGF, evaluating their mechanical and physicochemical properties, and their influence on the cultivation of mesenchymal stem cells. The future objective is to contribute to the increase of vascularization in models of GF controlled release, which can be applied in tissue engineering.

## Material and Methods

### Preparation of polymer solutions

The PLGA/BSA/VEGF scaffolds were produced by emulsification as follows: an organic phase, consisting of 15% PLGA (polylactic-co-glycolic acid) (w/w), 0.2% Span-80 and 1,1,1,3,3,3-hexafluoro-2-propanol (HFIP; Sigma-Aldrich, USA), and an aqueous phase consisting of 0.1% bovine serum albumin (BSA), 5% PBS and VEGF. The GF was added in the aqueous phase at a final concentration of 1 µg/mL. The PLGA/BSA scaffolds were constructed similarly, except for the presence of VEGF. The control group was produced with PLGA at a concentration of 13% (w/w) using HFIP (Sigma-Aldrich).

This study was approved by the Research Ethics Committee of the Hospital Santa Casa de Misericórdia de Porto Alegre (No. 3500/11).

### Electrospinning

The polymer scaffolds were produced by the electrospinning technique (ES). The voltage for the PLGA/BSA/VEGF and PLGA/BSA solutions was 18 kV, using an inner needle diameter of 0.80 mm and a flow rate of 0.28 mL/h. The distance between the needle and collector for both solutions was 20 cm. For the 13% PLGA solution, the voltage was 16 kV, using a 0.80 mm inner needle diameter with a flow rate of 0.28 mL/h and a distance between the needle and collector plate of 15 cm. The nanofiber scaffolds were adhered onto 1.5 cm^2^ diameter coverslips and sterilized with UV light for 1 h on a 24-well plate in a laminar flow hood.

### Human adipose derived stem cells (hADSCs) isolation and cell culture

hADSCs were obtained from elective liposuction procedures. Adipose tissue was subjected to enzymatic treatment with 0.075% collagenase type I at 37°C for 30 min. The hADSCs were seeded in culture bottles of 25 cm^2^. The medium was replaced after 24 h in order to isolate adherent cells, and thereafter it was refreshed once every 3 days to allow further growth; the cells were maintained at 37°C in a humidified atmosphere containing 5% CO_2_ until they reached 90% confluence. The cells were expanded to the fifth passage, at which the biological assays were performed.

### Characterization assays

Characterization consisted of differentiation in adipocytes, chondroblasts and osteoblasts, using a protocol already established in the laboratory (9). The immunophenotypic profile was analyzed using the antibodies CD14, CD34, CD44, CD45, CD73, CD90, CD146, CD184, Stro-1 and HLA-DR (Becton Dickinson, USA) conjugated with FITC or PE fluorochrome. Appropriate isotype controls were used and exclusion of dead hADSCs was performed by incubation with 7-aminoactinomycin D (7AAD). The analyses were performed on flow cytometer FACSAria III (Becton Dickinson) and analyzed by FACSDiva software, version 6.0. The graphs were created with WinMDI, version 2.8 (The Scripps Institutes - Flow Cytometry Core Facility, USA).

### Morphology, fiber diameter and pore size

The assessment of nanofiber morphology was performed by scanning electron microscopy (SEM), model - JSM 6060 (JEOL, Japan). The images were obtained using accelerating voltage of 10 kV. The diameters of the fibers were evaluated by ImageJ 1.38x software (NIH, USA) by measuring 30 fibers of each image obtained. The estimation of the pore size was performed using the same software. Ten pores of each image were analyzed by two measurements in opposite directions from each portion.

### Residual solvent content

The solvent content was evaluated by thermogravimetric analysis. The three groups of scaffolds were subjected to a heating ramp of 10° to 500°C/min. The equipment used was SDT Q600 (TA Instruments, USA).

### Degradability

The molecular weight of the degradation products was determined by gel permeation chromatography on the chromatograph Viscotek GPCmax VE2001 (Malvern Instruments, United Kingdom). The degradability test was conducted simulating physiological conditions at 37°C in PBS, pH 7.4. Approximately 30 mg of the scaffolds from each group were submerged in 10 mL of PBS at 120 rpm, at 37°C. Degradability was evaluated at the following times: 0 (scaffolds not subjected to degradability), 7, 14, and 30 days.

### Mechanical properties characterization

Young’s modulus, maximum load and elongation of the scaffolds were determined by dynamic mechanical analysis (DMA; n=5) (Q800AT DMA instrument equipped with a tension film clamp in the DMA controlled force mode, TA instruments, EUA). The scaffolds were cut in a rectangular shape (5×12 mm). The assays were carried out with constant temperature (37°C) with a ramp force 0.5 N/min to 18 N maximum load, under 0.005 N static load.

### Contact angle with water

Contact angle analysis was performed on five samples of each type of scaffold (PLGA/BSA/VEGF, PLGA/BSA, and 13% PLGA). A drop of water was instilled on the surface of the scaffold with the aid of a syringe and a photo was then taken at four different times: 5 and 30 s, and 1 and 2 min. Using the software Surftens 3.0 (OEG, Germany), at least three measurements of each picture were taken by using five measurement points arranged around the drop.

The contact angle of the films produced by the tested polymer solutions were also evaluated. For the production of polymer films, the different solutions were placed into petri dishes and subsequently stored for 24 h in a desiccator subjected to vacuum. In this experiment, a drop was instilled into three different points on the surface of each film, generating three images of each point. The assay was performed on four films of polymeric solutions, made on different days.

### Evaluation of VEGF incorporation efficiency

The PLGA/BSA/VEGF scaffolds were immersed in 30 mL of a dissolving solution (5 M urea; 0.1 NaOH; 0.08% SDS; 50 mM Tris) adapted from Sahoo et al. (10). They were maintained in agitation at 140 rpm for 3.5 h, with complete dissolution. After this period, all the volume was removed and the VEGF dosage was performed. Incorporation efficiency was calculated from the initial amount of VEGF added to the emulsion (1 µg of VEGF to 2 g of polymer solution), compared to the final amount of VEGF obtained after the dissolution of the matrices. Quantification of the VEGF concentration was performed by ELISA (ELISA Kit Human VEGF, Invitrogen®, USA) and absorbance was read in the spectrophotometer (450 nm) (Wallac EnVision, Perkin Elmer, USA).

### Evaluation of VEGF release

For evaluation of VEGF release from PLGA/BSA/VEGF matrices, approximately 200 mg (0.192±0.016 mg) of the scaffolds were immersed in 10 mL of DMEM containing 1% penicillin/streptomycin and fungizone. The same assay was performed for the PLGA/BSA scaffolds as negative control to evaluate the absence of a false positive detection of VEGF due to the constituents of the matrices. This was accomplished by placing the scaffolds under agitation at 120 rpm at 37°C. The concentration of VEGF was evaluated by ELISA (ELISA Kit Human VEGF, Invitrogen®) at intervals of 2, 8, 24, 72, 168, 336, and 504 h. In each of those periods, the total volume of DMEM was removed and the VEGF level was evaluated. The mediums were then renewed with 10 mL of DMEM and maintained under agitation, with subsequent measurements taken until the final time (504 h). Absorbance was analyzed identically to the VEGF incorporation efficiency test. The results are reported as percentage of cumulative release.

### Evaluation of HFIP solvent toxicity

In order to assess the potential toxicity of the solvent HFIP, 45,000 hADSCs were cultured per well and different concentrations of HFIP solvent (10, 50, 100, 250, and 500 ppm) in DMEM supplemented with 10% FBS and 1% antibiotic were evaluated. In addition, a control group without the addition of the solvent HFIP (0 ppm) was evaluated. After 72-h cultivation, 3-(4,5-dimethylthiazol-2-yl)-2,5-diphenyltetrazolium bromide assay (MTT; Sigma-Aldrich) was performed to verify cell viability. The tests were performed in triplicate (n=3).

### Cellular adhesion assay

For this assay, 30,000 hADSCs were seeded on different types of scaffolds and compared to the control group, which consisted of cells cultivated directly on the plastic surface of culture plates. Five different primary cultures of hADSCs were used and all the experiments were performed in triplicate. In the experiment, the cells were stained with 4.6-diamidino-2-phenylindole (DAPI), a marker of cell core, after 6 h of seeding on the scaffolds. Following this, photographs were taken in 9 points randomly chosen on the scaffolds and the average of cells per scaffold type was calculated. The result is reported as a mean number of cells.

### Cellular viability assay

Cell viability on the scaffolds was assessed by the colorimetric assay MTT (Sigma-Aldrich). The measurements were taken in triplicate on days 1, 4, 7, 14, and 21 after seeding the cells onto both the scaffolds and the wells (control group; n=5). After each time point, the cells were incubated with 0.25 μg/mL MTT for 2 h. Four hundred microliters of dimethylsulfoxide (DMSO) was then added per well to dissolve the crystals formed. Absorbance was measured at 560 and 630 nm. The results were calculated by the absorbance label subtraction (560–630 nm) and reported as the average absorbance for each group. The absorbance measurement was performed with the Wallac EnVision equipment (Perkin Elmer).

### Cytotoxicity assay

Cytotoxicity was assessed by measurement of the enzyme lactate dehydrogenase (LDH; Labtest, Brazil). Aliquots of the supernatant of the cultures were measured after 4, 7, 14, and 21 days of cultivation. As negative control, the cells cultured directly on the wells were used. The positive control consisted of cells on the wells treated with 1% (v/v) Triton X-100 (Sigma-Aldrich) for 10 min. Absorbance was measured using the equipment Wallac EnVision equipment (Perkin Elmer).

### Statistical analysis

The results for the adhesion assay, cytotoxicity and cell viability are reported as means±SE. The other results are reported as means±SD. The cell viability, the cytotoxicity assay and the contact angle were evaluated by repeated measures analysis with two factors: time and group (Hotelling) and Bonferroni post-test. Adhesion tests were evaluated by ANOVA (one-way) and Tukey’s test was used for the post-test. The results were generated by SPSS 16 software (IBM, USA).

## Results

**Figure 1.**
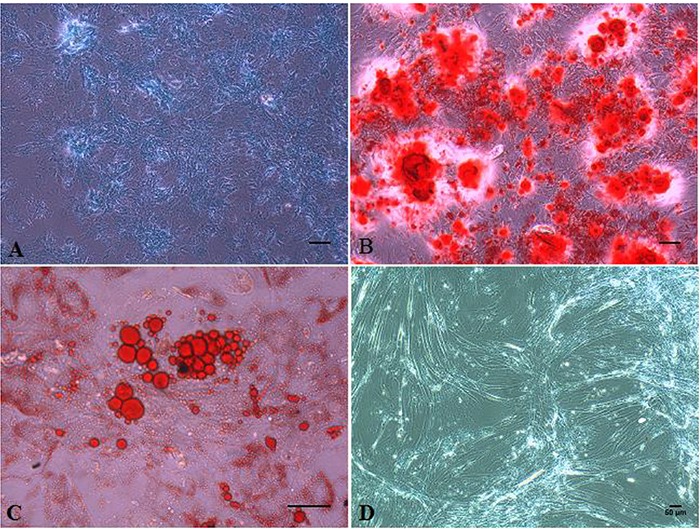
Differentiation of the human adipose derived stem cells. *A*, Condrogenic differentiation with Alcian Blue dye; *B*, osteogenic differentiation with Alizarin Red dye; *C*, adipogenic differentiation with Oil Red dye; *D*, non-differentiated cells not submitted to dying with typical morphology of mesenchymal stem cells. Images *A*, *B* and *D* with 200× magnification and image *C* with 400× magnification. Scale bars: 50 µm.

The cells obtained through liposuction aspirates were isolated, cultivated and characterized successfully. They presented typical morphology of mesenchymal stem cells (MSCs) in cultivation *in vitro* and were differentiated with success into adipocytes, chondrocytes and osteocytes (Figure 1). The cells presented high positivity for the following expressions: CD29 (98.2±1.1%), CD44 (76.65±2.55%), CD73 (83.2±15%), and CD90 (90.05±8.65%). Some antigens presented low expression: CD34 (3.15±1.75%), CD184 (0.6±0.6%), and STRO-1 (2.8±2.5%) and others presented negative expression: CD14, CD45 and HLA-DR.

**Figure 2.**
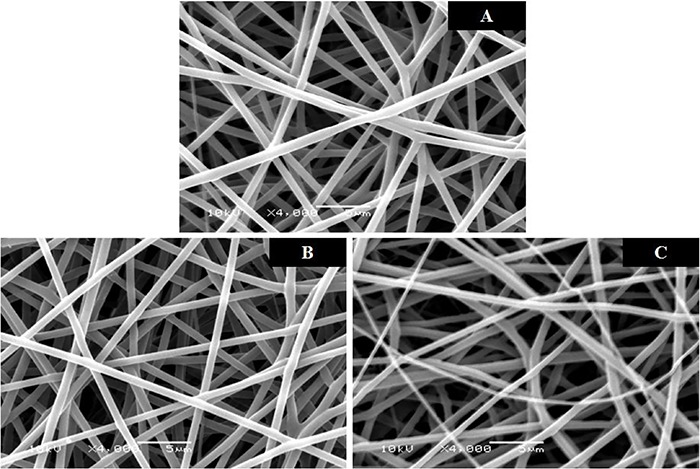
Microphotograph of the morphology of the scaffolds using scanning electron microscope (4,000× magnification). *A*, PLGA/BSA/VEGF matrix; *B*, PLGA/BSA scaffold; *C*, PLGA 13% matrix. PLGA: polylactic-co-glycolic acid; BSA: bovine serum albumin; VEGF: vascular endothelial growth factor.

The three groups of scaffolds presented well-formed and randomly distributed fibers without beads and with interconnected pores (Figure 2). The diameters of the fibers were 588±123 and 554±164 nm for the PLGA/BSA/VEGF and 13% PLGA matrices, respectively. The PLGA/BSA group presented an average fiber diameter of 520±131 nm and were statistically different from the other groups with P<0.05 for the PLGA/BSA/VEGF scaffolds and P<0.001 for the 13% PLGA scaffolds.

The values for the pore size were 4.96±1.78, 4.56±2.03, and 5.01±2.19 μm for the PLGA/BSA/VEGF, PLGA/BSA and 13% PLGA scaffolds, respectively. No statistical difference was observed among the groups (P=0.551).

**Figure 3.**
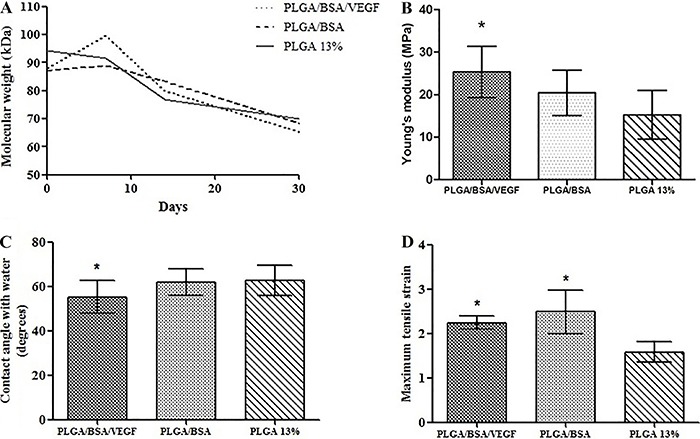
*A*, Loss of molecular weight evaluated by gel permeation chromatography at different periods: 0 (not submitted to the degradation process), 7, 14, and 30 days (PLGA/BSA/VEGF, PLGA/BSA and 13% PLGA matrixes) (repeated measures with *post-hoc* Bonferroni). *B*, Average Young’s modulus for the different scaffold groups (one-way ANOVA with *post-hoc* Tukey’s test). *C*, Contact angle in the PLGA/BSA/VEGF, PLGA/BSA and 13% PLGA films with average values of 55.22±3.22°; 61.72±4.3° and 62.73±4.66°, respectively (repeated measures with *post-hoc* Bonferroni). *D*, Maximum tensile strain for the different scaffold groups (one-way ANOVA with *post-hoc* Tukey’s test). Data are reported as means±SD. *P<0.05 compared to 13% PLGA. PLGA: polylactic-co-glycolic acid; BSA: bovine serum albumin; VEGF: vascular endothelial growth factor.

During the 30 days of analysis, there was a degradation rate of approximately 25% for the PLGA/BSA/VEGF group, 22% for the PLGA/BSA group and 13% for the 13% PLGA matrices. There was no statistical difference in the temporal analysis between the groups at any period of evaluation (P= 0.956; Figure 3A).

The contact angle was evaluated in the three types of scaffold produced by ES. The results obtained are shown in Table 1.

**Table 1.** Measurements of the contact angle in the PLGA/BSA/VEGF, PLGA/BSA and 13% PLGA at different time periods in seconds (n=4).

	Contact angle (^°^)			
	5 s	30 s	60 s	120 s
PLGA/BSA/VEGF	121.12±4.4	89.8±6.5	126.22±5.11	121.6±661
PLGA/BSA	103.3±6.26	102.7±7.57	101±8.64	98.7±8.94
PLGA (13%)	108.6±2.61	107.21±2.72	107.25±2.84	105.7±3.4

PLGA: polylactic-co-glycolic acid; BSA: bovine serum albumin; VEGF: vascular endothelial growth factor.

The contact angle was also analyzed in polymeric film produced from the PLGA/BSA/VEGF, PLGA/BSA emulsions and the 13% PLGA polymeric solution. The three types of film produced presented hydrophilic profiles with contact angles below 90°. The films produced from the PLGA/BSA/VEGF solution presented greater hydrophilic profiles with a contact angle of 55.22±3.22°. This was statistically lower (P=0.037) than the angle encountered in the PLGA 13% film, which was 62.73±4.66°. The PLGA/BSA films presented an intermediate contact angle of 61.72±4.3°, which did not demonstrate a statistical difference in relation to the other groups (P>0.05 for all comparisons; Figure 3B).

Young’s modulus and the maximum tensile strain of the PLGA/BSA/VEGF scaffolds were 25.3±5.59 and 2.2±0.13, respectively. The PLGA/BSA scaffolds presented an average Young's modulus of 20.4±5.02 and maximum tensile strain of 2.4±0.44. The 13% PLGA scaffold group presented values of 15.41±5.41 and 1.5±0.20 for Young’s modulus and maximum tensile strain, respectively. Rupture did not occur in any of the samples.

The values were greater for both Young’s modulus and maximum tensile strain in the PLGA/BSA/VEGF scaffolds compared to the 13% PLGA control (P=0.005 and P=0.027, respectively). The PLGA/BSA scaffolds presented intermediate values for Young’s modulus with no statistical difference in relation to the other groups; however, this same group presented a maximum tensile strain greater than the 13% PLGA group (P=0.01) and obtained no statistical differences in relation to the PLGA/BSA/VEGF scaffolds (P=0.525) (Figure 3C and D).

**Figure 4.**
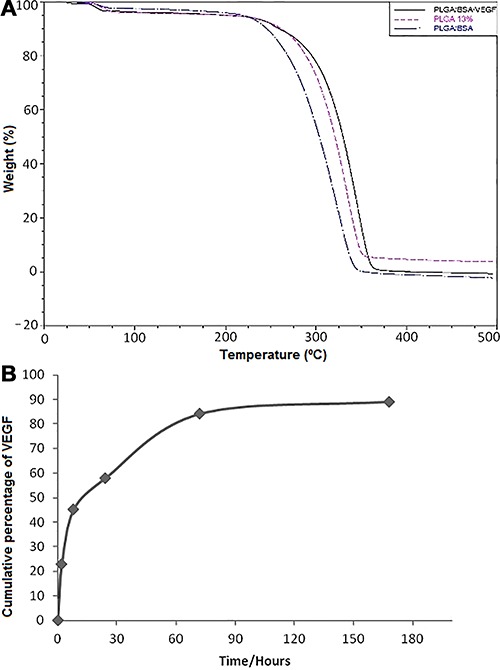
*A*, Graph obtained by thermogravimetric analysis showing the mean percentage of weight loss of the PLGA/BSA/VEGF, PLGA/BSA and PLGA 13% scaffolds. *B*, *In vitro* VEGF liberation curve from the PLGA/BSA/VEGF scaffolds. PLGA: polylactic-co-glycolic acid; BSA: bovine serum albumin; VEGF: vascular endothelial growth factor.

Verification of the residual solvent content was performed using thermogravimetric analysis. The results of this analysis can be observed in Figure 4A. The weight loss in relation to the three scaffold groups occurred between 50° and 75°C. The values for weight loss and the residual solvent content can be seen in Table 2.

**Table 2.** Average quantity of residual solvent in the various scaffolds (n=3).

Scaffolds	Residual solvent	
	Weight loss (%)	Concentration of HFIP (ppm)
PLGA/BSA/VEGF	2.46±0.21	14.5±1.23
PLGA/BSA	1.5±0.22	10.4±1.52
PLGA 13%	3±0.45	29.6±4.44

PLGA: polylactic-co-glycolic acid; BSA: bovine serum albumin; VEGF: vascular endothelial growth factor; HFIP: 1,1,1,3,3,3-hexafluoro-2-propanol.

The incorporation rate of the growth factor into the PLGA/BSA/VEGF scaffold was 3.47±0.96% (n=3). Of the total amount of VEGF incorporated in the fiber, there was an average liberation of 91.37% in a period of 504 h (21 days; n=3). A liberation burst was observed in the first 8 h, with a steep increase up to 72 h when it reached a plateau (Figure 4B).

**Figure 5.**
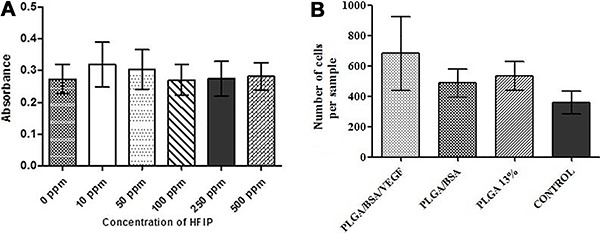
*A*, Evaluation of the metabolically active cells by MTT after cultivation in a DMEM medium with various concentrations of 1,1,1,3,3,3-hexafluoro-2-propanol (HFIP) solvent (repeated measures with *post-hoc* Bonferroni). *B*, Cellular adhesion after 6 h of cultivation *in vitro* for 5 primary cultures of human adipose derived stem cells tested on the following scaffold groups: PLGA/BSA/VEGF, PLGA/BSA, 13% PLGA and the control (cells cultivated directly on the cultivation plate wells) (one-way ANOVA with *post-hoc* Tukey’s test). Data are reported as means±SE. PLGA: PLGA: polylactic-co-glycolic acid; BSA: bovine serum albumin; VEGF: vascular endothelial growth factor.

After 72 h of cultivation of the hADSCs in a culture medium containing HFIP solvent, no statistical difference was observed (P=0.929) in the various concentrations of the tested HFIP (Figure 5A).

The cells demonstrated a higher adhesion tendency although there was no statistical difference (P=0.490) in the PLGA/BSA/VEGF scaffolds compared to the other two scaffold groups (PLGA/BSA e PLGA 13%) and the control (cells cultivated directly on the wells) (Figure 5B).

**Figure 6.**
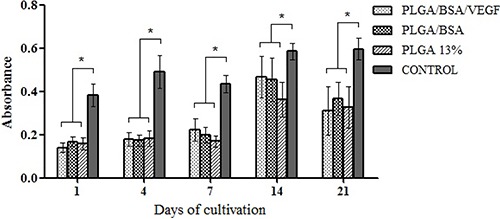
Evaluation of the metabolically active cells by MTT assay, tested on the following scaffold groups: PLGA/BSA/VEGF, PLGA/BSA, 13% PLGA and the control (cells cultivated directly on the cultivation well plates). Data are reported as means±SD. *P<0.05, repeated measures with *post-hoc* Bonferroni. PLGA: polylactic-co-glycolic acid; BSA: bovine serum albumin; VEGF: vascular endothelial growth factor.

Cell viability for the PLGA/BSA/VEGF, PLGA/BSA and 13% PLGA groups over the 5 evaluated periods did not present any statistical differences (P=0.916). The control group presented the highest number of viable cells compared to the other groups for all the periods of analysis (P<0.001; Figure 6). However, between the 21st and the 1st day of analysis, the following proliferation rates for the scaffolds were observed: 2.23 for the PLGA/VEGF/BSA, 2.16 for the PLGA/BSA, 2.05 for the 13% PLGA and 1.55 for the control.

**Figure 7.**
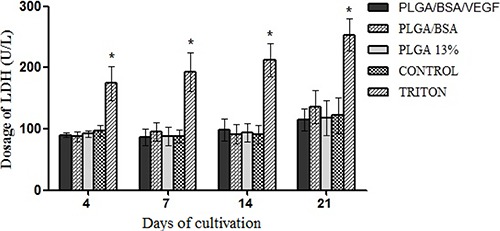
Dosage of lactate dehydrogenase (LDH) in supernatant of primary cultures of human adipose derived stem cells tested on the following scaffold groups: PLGA/BSA/VEGF, PLGA/BSA, 13% PLGA, negative control group (cells seeded directly on the cultivation well plates) and the positive control group (Triton). Data are reported as means±SD. *P<0.05 compared to 13% PLGA (repeated measures with *post-hoc* Bonferroni). PLGA: polylactic-co-glycolic acid; BSA: bovine serum albumin; VEGF: vascular endothelial growth factor.

The dosage of LDH for the three scaffold groups were similar to the negative control group (P=1.000 for all comparisons). The scaffolds continued to present dosages of LDH well below the Triton 1% group (positive control), which present a maximum liberation of LDH, or, in other words, total cell death (Figure 7; P<0.003 for all comparisons).

## Discussion

The choice of the biomaterial to be used in TE must meet certain fundamental characteristics to achieve success. The biomaterial should be both biocompatible and biodegradable, and able to support cellular adhesion and proliferation (11). It also must deliver growth factors, which are important for tissue regeneration, permit the diffusion of nutrients through the biomaterial and have degradation kinetics compatible with the location of regeneration. It should also present a tridimensional structure that can mimic the extracellular scaffold (ECM) (12-14) and be capable of supporting cellular adhesion and proliferation, facilitating the contact between cells (13,15).

A further important process for TE is angiogenesis, a complex and dynamic event that depends on the interaction of various factors, including cell type, growth factors, extra cellular scaffold and the biomechanical properties of the matrices (16). Therefore, various properties of the developed scaffolds that are important for the success of TE have been analyzed in this study.

Despite finding statistical differences between the diameters of the PLGA/BSA scaffolds in relation to the other groups, it is believed that there is no relevant physiological difference in relation to the average diameter of the fibers. The values are very similar and all groups presented fibers on a nanometric scale, similar to the ECM, the diameters of which range from 100 to 600 nm (17). When in contact with the cells, structures with the aforementioned characteristics allow for an elevated cellular organization (18), reduction of the apoptosis and necrosis, and continued cell functionality (19). Furthermore, the nanofibers presented an increased number of interconnected pores, as can be observed in the SEM images, ensuring the tridimensional arrangement, which can not only favor cellular migration but also guarantee the exchanges of nutrients and metabolites with the external environment (20).

The size, number and interconnectivity of the pores of the scaffolds are important factors in the determination of their compatibility with the cells (21). Pore sizes with variances of micrometer to millimeter scale greatly affect cell migration because extremely large pores prevent contact of the cells with the biomaterial. In contrast, pores which are under 100 nm have a negative influence on the diffusion of nutrients and oxygen, and the elimination of residues. Therefore, inadequate diffusion of nutrients can result in a reduction of cell survival and the porosity of the biomaterial must be balanced to obtain good integration with the cells (21). An analysis of the pore size of the three scaffold groups showed that they were adequate for cell cultivation because they presented intermediate pore sizes on a micrometric scale, allowing an efficient delivery of nutrients and oxygen, and elimination of residues.

Organic solvents, such as HFIP used in the production of nanofiber matrices, may be harmful for the cells (22,23). The content of the residual solvent was therefore evaluated to determine whether the scaffolds produced by electrospinning can present any harmful effects for cellular development. Analysis of the toxicity results obtained from the cultivation of hADSCs with HFIP in the various concentrations did not reveal any statistical difference between the groups (10, 50, 100, 250, and 500 ppm) and the control (0 ppm), demonstrating that higher concentrations of HFIP did not result in toxicity for the cells. In this study, the values of the solvents were 14.5, 10.4, and 29.6 ppm for the PLGA/BSA/VEGF, PLGA/BSA and 13% PLGA matrices, respectively. Low concentrations of residual HFIP were found in the nanofiber matrices, the values being well below 500 ppm, which was the largest concentration tested in cellular assay with HFIP. When making a correlation between this finding and the cytotoxicity test with LDH dosage, it was observed that there was no negative effect for the cells in all the analyzed matrices. It was also observed that the dosages of LDH in all the scaffold groups were very similar to the negative control. These results corroborate with the results of Nam et al. (24), who evaluated the quantity of residual HFIP in the PCL scaffolds produced by ES. This study demonstrated that values of up to 250 ppm of this solvent did not result in toxicity for the chondrocytes *in vitro* (24).

Degradation is an important point that should be evaluated in accordance with the regeneration site. Ideally, the degradation rate of the biomaterial should be similar to the regeneration rate of the tissue (25). In this study, the degradation of the three types of scaffold was approximately 25% in 30 days. It should be taken into consideration that *in vivo* the degradation rate would probably be higher because other plasma components could accelerate the process (26). Consequently, it is believed that the produced scaffolds could be good candidates for tissue regeneration, functioning as release devices controlled by GFs, because after the initial burst (GFs probably present on the surface), the liberation of the bioactive agents occurs when the scaffolds are being degraded (27). If the scaffolds have slow degradation, the GF will not be released in a timely manner to promote tissue regeneration.

Evaluation of the hydrophilicity/hydrophobicity of the scaffolds is made by the measurement of the contact angle. The materials are considered hydrophilic when the measurement of the contact angle is lower than 90° and hydrophobic when it is above 90°. Studies have demonstrated that moderate contact angles with values between 40° and 70° are ideal for cellular adhesion (28). The contact angle measurement was made in the three scaffold groups and also in polymeric films produced from the same polymeric solutions. In the temporal analysis, the three groups demonstrated a hydrophobic profile in practically all the evaluation time periods, with the exception of the 30-s period for the PLGA/BSA/VEGF matrices, which had an angle consistent with the hydrophilic material. However, this difference appears to be random because it did not occur in the sequential time periods, in which once again a hydrophobic profile was observed. Some studies have demonstrated that scaffolds produced from emulsions present a significant reduction in the contact angle measurement, which was not observed in this study (29,30). One hypothesis for this fact is that materials with porous and rough structures, such as the scaffolds produced by electrospinning, present hydrophobic characteristics due to the large quantity of air present in their pores (31,32). The results obtained through the contact angle measurements in films made from the same polymeric solutions corroborate with this hypothesis, remembering that in this case, the films presented hydrophilic characteristics. Because greater hydrophilicity was observed in the PLGA/BSA/VEGF films, it is suggested that the scaffolds produced in this group present better results for cellular adhesion as, according to former studies, the cells present greater adhesion and growth in hydrophilic materials, which are, therefore, the most highly recommended for use in biomedical applications (33–35).

The mechanical characteristics of the biomaterials are determined by both the properties of the actual material and its structure. The interaction of the mechanical properties of the materials with the graft setting is of utmost importance for the progression of tissue regeneration not to be limited by mechanical failures of the biomaterials and for tissue regeneration to occur (7,36). In this study, the scaffolds produced from emulsion presented a greater Young’s modulus and also greater maximum stress and strain. These scaffolds were shown to be more resistant than the 13% PLGA scaffold. It is suggested that the increase in tensile strength in the scaffolds produced from emulsion, should be related to the presence of nanoparticles of Span-80 contained in the emulsion, which may have become attached to the nanofibers, acting as a re-enforcement to their resistance. This result is corroborated by a previous study of Li et al., in which an increase occurred in Young’s model in the scaffolds produced from emulsion containing the surfactant Span-80 (29).

In relation to the incorporation rate of the GF on the matrices, the results obtained were 3.47±0.96% (n=3). A similar finding was observed by Chew et al., who encountered incorporation efficiency results of 3.10% for the neural growth factor (37). Another study using PLGA scaffolds produced from emulsion, presented incorporation efficiency of 1.56% for the BSA (38). The low efficiency results may have occurred due to the physicochemical instability of the VEGF during the various processes for the production of the scaffolds (27). Initially, the methodology itself presented a loss of components during its execution, attested by the formation of fibers outside the collecting plate. Besides, the production of the fibers involves stages, which can contribute to the loss of bioactivity of the protein because of the alterations in the spatial conformation of the protein structures in contact with the polymeric solution. The mechanical homogenization process of the emulsion, therefore, along with the contact with the organic solvent and the high tension to which the polymeric solution is submitted during the fabrication process of the scaffold should be highlighted. There are some strategies that have the aim of minimizing the effects described above and which guarantee the bioactivity and functionality of the GF. Among these is the addition of surfactants, such as Span-80, used with the objective of internalizing the aqueous fraction containing the protein and therefore lessening the contact with the organic solvent (29). The addition of other proteins, such as albumin, has already been described as a stabilizer of GFs during the ES process. In the present study, these strategies were adopted to minimize the effects of ES on the VEGF. Initially, albumin was added to the aqueous solution with the objective of reducing the instability of the bioactive molecule. Together with this, the surfactant Span-80 was added during the oil phase of the emulsion to lessen the exposure of the VEGF to the organic solvent, HFIP. However, as was observed, these strategies had little effect in obtaining a high incorporation value of the GF. A point highlighted in the work of Chew et al. (37) to explain the low level of incorporation encountered, which is also applicable in the results found in the present study, is the fact that the electrostatic forces work in a different way in the two phases of the polymeric solution, ejecting, therefore, the two phases with different velocities and making it impossible to incorporate the GF with the nanofibers (37).

However, it can be emphasized that during tissue regeneration using MSCs, there is a paracrine action of the latter with liberation of growth factors, among them being VEGF. According to Parekkadan et al. (39), 10^6^ MSCs *in vitro* produce approximately 100 pg GF in 24 h. From this, it is estimated that to obtain 10,000 pg GF from MSCs, 100 million cells would be necessary. In the present study, the average incorporation efficiency in the PLGA/BSA/VEGF scaffolds was approximately 3.5%, or 35,000 pg. Considering the aforementioned facts, physiologically, the quantity of VEGF present in the polymeric scaffold would be equivalent to an infusion of 350 million MSCs (39). However, despite the low incorporation efficiency rate, the VEGF incorporated in the scaffold could be sufficient to obtain a physiological result, which would in turn be complemented with a local cellular secretion.

On the other hand, the PLGA/BSA/VEGF scaffolds appeared to be good devices for the controlled liberation of GFs. From the total VEGF incorporated in the fiber, there was an average liberation of 91.37% in a period of 504 h (21 days) (n=3). A burst was observed in the first 8 h, with liberation increases up to 72 h, when it appeared that a plateau was established (Figure 4B).

Cellular adhesion is the first step in a sequence of events that direct the cellular and biomaterial interaction. The biomaterials should promote cellular adhesion, which is a fundamental characteristic for cell survival, because the absence of this contact can initiate mechanisms of programmed cell death (40). All the scaffold groups presented high cell adhesion values compared to the control group. Despite the fact that there was no statistical difference among the four evaluated groups, a tendency for greater cell adhesion was observed in the PLGA/BSA/VEGF group. This behavior could be related to the fact that the films produced from the PLGA/BSA/VEGF polymeric solution presented a higher hydrophilic profile and were also more adept for cellular adhesion. Furthermore, the GFs have a leading role in cell behavior. Adhesion, proliferation and differentiation are to a greater extent dictated by the presence of GFs, which are soluble in the ECM (17). The VEGF present in the scaffolds may have increased the bioactivity of the PLGA/BSA/VEGF scaffold and, consequently, supported cellular adhesion in the latter.

Although the use of emulsions is widely discussed in the literature, there are still questions concerning the possible negative effect on the growth of cells cultivated in scaffolds containing surfactants, as for example Span-80 (29). Cells cultivated on scaffolds produced by emulsion present similar growth to the 13% PLGA control group prepared with just the PLGA polymer dissolved in HFIP. The proliferation rate was very similar amongst all the groups, including the control (cells seeded directly onto the wells). This latter group presented the lowest proliferation rate compared to the other groups. It can be concluded, therefore, that cells seeded on the scaffolds performed better compared to the control; however, because of the longer period required for the adaption of the latter with the polymeric matrices, cells did not reach the same level as the cells that were seeded directly on the culture wells in the same period of analysis. Similar results were found by Steffens et al. (19) when cultivating MSCs on PDLLA and PDLLA/*Spirulina* scaffolds.

Regarding viability in relation to the time of cultivation, it was observed that there was a higher number of metabolically active cells on the 14th day (P<0.001) compared to the other time periods, with the exception of the 21st day of analysis, in which there was no statistical difference (P=1.000). Through microscope analysis, saturation of the well culture could be observed after the 14th day of cultivation. In other words, the cells no longer possessed physical space for their multiplication.

In the present study, cellular toxicity was evaluated through dosages of the enzyme LDH, which is found in practically all organs and tissue of the organism. The increase in the extracellular concentration of this enzyme is proportional with the increase in cell death (19). In this assay, it was observed that the three types of scaffolds did not have cytotoxicity for the hADSCs because all presented much lower dosages of LDH compared to the positive control. During the period of analysis, the highest dosage level of the enzyme LDH (P<0.05) was found on the 21st day, suggesting greater cell death. This finding can be correlated with the reduction of cellular metabolism between the 14th and 21st day of cellular cultivation, which was observed by the MTT assay, as well as the greater number of cells, when compared to the other days of analysis.

The scaffolds developed from emulsion presented well-distributed, smooth fibers on a nanometric scale with a porous structure similar to that of the ECM of the cells. They presented appropriate characteristics for cell cultivation when applied to TE. These scaffolds have been shown to be safe for pre-clinical use because scaffolds produced by emulsion ES with or without VEGF have been shown to be non-toxic for stem cells. A proliferation rate has also been verified, which was similar among the three scaffold groups with the PLGA/BSA/VEGF scaffold presenting a higher proliferation rate, suggesting a contribution from the GFs. Furthermore, the scaffolds degraded during the period of study, which is an ideal characteristic for release devices applied to TE.

The scaffolds containing VEGF presented liberation supported by GFs, which is a fundamental requisite for materials for *in vivo* implantation. Despite the efficiency of the GF incorporation by the emulsion electrospinning technique not being high, it is hoped that the quantity of VEGF incorporated in the scaffolds will be sufficient to contribute to tissue regeneration because these molecules operate in greatly reduced concentrations in the organism. It is recommended that this be clarified by *in vivo* assays.
